# Flexible Low-Dose GnRH Antagonist Protocol Is Effective in Patients With Sufficient Ovarian Reserve in IVF

**DOI:** 10.3389/fendo.2018.00767

**Published:** 2018-12-19

**Authors:** Dan Zhang, Lan Xia, Huihui Xu, Qian Chen, Bailing Jin, Aijun Zhang, Bufang Xu

**Affiliations:** ^1^Reproductive Medical Center of Ruijin Hospital, School of Medicine, Shanghai Jiao Tong University, Shanghai, China; ^2^Shanghai Key Laboratory of Reproductive Medicine, Department of Histo-Embryology, Genetics and Developmental Biology, Shanghai Jiaotong University, School of Medicine, Shanghai, China

**Keywords:** flexible low-dose GnRH-ant protocol, Cetrorelix, IVF, LH surge, GnRH antagonist

## Abstract

Gonadotropin-releasing hormone antagonist (GnRH-ant) has been shown to negatively influence endometrial receptivity. Reducing the GnRH-ant dose during controlled ovarian stimulation (COS) when using a GnRH-ant protocol may be beneficial to embryo implantation. However, whether or not the minimum daily GnRH-ant dose should be individualized remains uncertain. In this retrospective study, we aimed to elucidate the feasibility and effectiveness of moderately reducing the daily GnRH-ant dose to 0.125 mg, and then adjusting the dose to 0.25 mg based on subsequent luteinizing hormone (LH) levels. Of the 434 patients analyzed in this study, 209 received our new flexible low-dose GnRH-ant protocol (Group 1) and 225 received a conventional GnRH-ant protocol with a fixed daily dose of 0.25 mg (Group 2). Furthermore, 105 and 114 cycles from groups 1 and 2 received fresh embryo transfer. In Group 1, 30 patients whose dose of 0.125 mg GnRH-ant was adjusted according to their LH levels and 179 patients who received consistently low doses were further divided into subgroups 1 and 2, respectively. Neither the number of retrieved oocytes and available embryos nor the implantation rate, clinical pregnancy rate, and ongoing pregnancy rate significantly differed between the two groups. However, GnRH-ant dose and stimulation duration were much lower and shorter in Group 1 than in Group 2 (*p* < 0.05). Subgroup 1 exhibited higher basal follicle-stimulating hormone (FSH) and lower antral follicle count (AFC) than subgroup 2 significantly. The number of retrieved oocytes and available embryos were lower in subgroup 1 than in subgroup 2 (6.83 ± 3.28 vs. 11.83 ± 4.82, 2.93 ± 1.86 vs. 4.99 ± 3.46, respectively, *p* < 0.05), while more canceled cycles for pre-ovulation occurred in subgroup 1 than in subgroup 2 (3/30 vs. 1/179, *p* < 0.05). The results showed that the flexible low-dose GnRH-ant protocol was as effective as the conventional fixed-dose GnRH-ant protocol with 0.25 mg per day for most patients with normal ovarian reserve. This retrospective analysis and the small sample size are the main limitations of this study, and a large sample RCT will be carried out in the future.

## Introduction

The gonadotropin-releasing hormone antagonist (GnRH-ant) protocol has been widely used in *in vitro* fertilization-embryo transfer (IVF-ET) for more than 15 years. Compared with the GnRH agonist long protocol, it is known to have several advantages, including shortened treatment duration, lower gonadotropin requirement, avoidance of excessive pituitary suppression and flare-up side effects, and a reduction in the incidence of severe ovarian hyperstimulation syndrome (OHSS) (1). Although the live birth rate achieved with GnRH-ant protocol has been reported to be comparable to that achieved with GnRH-agonist protocol (2, 3), other studies reported a lower pregnancy rate with the GnRH-ant protocol (4, 5). A series of recent studies has confirmed that the adverse effects of GnRH-ant on endometrial receptivity were the main reason for this difference in pregnancy rate (4). Furthermore, in our previous studies we demonstrated the dose-related harmful effects of GnRH-ant on endometrial receptivity (6, 7).

To minimize the harmful effects of GnRH-ant to the endometrium, clinical researchers have attempted to determine the minimum GnRH-ant dose to improve the GnRH antagonist protocol. Thus far, the use of 0.25 mg of Cetrorelix daily from day 6 of stimulation has been considered capable of maintaining the LH level within the safe range, and this dosage is commonly used in clinical practice (8–11). Regardless, few studies have reported that reducing the GnRH-ant dose to as little as 0.125–0.2 mg per day is also equally effective (12–14). Wang et al. even reported that routine GnRH-ant administration was not required, as 87.2% of the patients did not need any antagonist throughout the stimulation cycle (15). Thus, the minimum possible dose of GnRH antagonist that is suitable for IVF-ET remains controversial. We assume that there is no specific GnRH-ant dose that would suit everyone, and adjusting the dose individually is therefore necessary. Based on the premature LH surge that defined as LH level ≥10 IU/L and progesterone level ≥1.0 ng/ml (16, 17), and the effective low GnRH-ant dose reported by previous papers, we tried a new flexible low-dose GnRH-ant protocol in some patients in which the daily GnRH-ant dosage was reduced to 0.125 mg and adjusted to 0.25 mg if the LH level reached 10 IU/L until the day before trigger day.

## Materials and Methods

### Patients

In this retrospective study, we obtained data of patients who received the GnRH-ant protocol for IVF from January 2016 to June 2018 at the Reproductive Medical Center of Ruijin Hospital, School of Medicine, Shanghai Jiao Tong University. Ethical approval was obtained from the Institutional Ethics Committee of Ruijin Hospital, School of Medicine, Shanghai Jiao Tong University. Approval from the institutional review board was obtained for the analysis of this series. Patients who underwent GnRH-ant protocols for the first time were selected for this study.

### Inclusion/Exclusion Criteria

The inclusion criteria were as follows: (a) 20–45 years old, (b) signed informed consents, (c) basal serum FSH < 15 IU/L (16). The exclusion criteria were as follows: (a) chromosomal aberration in either the mother or father, (b) serum LH > 10 IU/L on the GnRH-ant start day, or (c) history of repeated IVF.

### Groups According to Different GnRH-Ant Protocols

Patients were divided into two groups according to the usage of Cetrorelix: Group 1 (*n* = 209) initially received 0.125 mg of Cetrorelix per day. The patients underwent routine observation every 1–2 days, and the daily Cetrorelix dose was adjusted to 0.25 mg once the serum LH level exceeded 10 IU/L. The patients in Group 1 were further divided into two subgroups depending on whether the Cetrorelix dose was increased (subgroup 1) or remained unchanged (subgroup 2) for these patients. The Cetrorelix dose was increased to 0.25 mg per day in 30 patients while it remained unchanged for 179 patients; these were designated as subgroups 1 and 2, respectively. A total of 225 patients in Group 2 received 0.25 mg of Cetrorelix per day from the beginning to the end. Nothing else was changed during COS between the two groups except for the Cetrorelix usage.

### IVF-ET Procedure

The stimulation was initiated on menstrual day 2, and all the patients were administered recombinant FSH (Gonal-F, Merck-Serono SA, Switzerland). The initial gonadotropin dose was experientially determined by doctors according to age, antral follicle count (AFC), basal FSH, E_2_ levels, and body mass index (BMI), and typically ranged from 150 to 300 U per day. This dose was adjusted every 2–3 days of stimulation depending on the ovarian response evidenced by the E_2_ levels and follicular growth detected under ultrasound examination. Then, all the patients received the GnRH-ant Cetrorelix acetate (Cetrotide, Merck-Serono SA, Switzerland) from the sixth stimulation day to the day before trigger day. Finally, 3,000–5,000 IU of human chorionic gonadotropin (hCG; Lizhu, Zhuhai, China) was administered when three follicles reached a mean diameter of 17 mm. Oocyte retrieval was performed 35–36 h after hCG injection by transvaginal ultrasound-guided single-lumen needle aspiration. Intracytoplasmic sperm injection (ICSI) was performed only in case of severe male factor infertility. Oocyte culture, insemination, embryo transfer, and cryopreservation were done as previously described (17). Embryo transfer was conducted on day 3 after oocyte retrieval. All the patients received embryo transfer on day 3, except in the following cases: (a) serum estrogen >7,000 pg/mL on the trigger day, (b) more than 15 oocytes were retrieved, (d) the presence of uterine or endometrial abnormalities such as endometriosis, uterine myoma, endometrial polyps, or intrauterine adhesion, (e) an initial increase of progesterone over 1.5 ng/mL before the trigger day (18), or (f) the patient refused fresh embryo transfer. A maximum of two embryos were transferred. The luteal phase was supported by 90 mg of sustained-release progesterone gel (8% Crinone; Merck-Serono, Switzerland) administered vaginally from the first day after oocyte retrieval. Clinical pregnancy was determined by visualizing a gestational sac on ultrasound at 6 weeks of gestation. Ongoing pregnancy was defined as a viable pregnancy beyond 12 weeks of gestation (19).

### Statistical Analysis

Data were analyzed using SPSS version 18.0 (IBM). Frequency for qualitative variables, and the means and standard deviation for quantitative variables were calculated. The chi-square test and Fisher's exact test and the Student's *t*-test for independent samples were used. Statistical significance was defined as *p* < 0.05.

All the raw data and analysis have been included as Supplementary Files.

## Results

Table 1 presents the demographic characteristics of both groups. There were no significant differences in the baseline characteristics, including age; BMI; AFC; and basal FSH, LH, and E_2_ levels between the two groups.

**Table 1:** Demographic characteristics of participants.

**Groups**	**Group1 (*n* = 209)**	**Group2 (*n* = 225)**	***p***
Age (years)	29.92 ± 4.34	30.48 ± 4.41	0.179
Body mass index	21.82 ± 3.44	21.89 ± 3.74	0.842
Basal FSH level (IU/L)	7.47 ± 1.87	7.39 ± 1.79	0.653
Basal LH level (IU/L)	4.56 ± 2.28	4.31 ± 2.01	0.236
Basal E_2_ level (pg/ml)	33.78 ± 15.56	34.40 ± 17.07	0.696
Antral follicle count	12.94 ± 5.65	12.73 ± 5.65	0.706
**INFERTILITY CAUSES**			
Primary infertility	140 (67%)	148 (65.8%)	0.790
Male factor alone	101 (48.3%)	113 (50.2%)	0.693
Tubal factor alone	146 (69.8%)	153 (68%)	0.676
Ovulation disorders	32 (15.3%)	30 (13.3%)	0.556
Endometriosis	16 (7.66%)	18 (8%)	0.894
Other^*^	3 (1.43%)	3 (1.33%)	0.927

* *Unexplained infertility after 2 or more times of intrauterine insemination. Data expressed as mean ± SD, or number (percentage)*.

As shown in Table 2, Cetrorelix consumption was significantly lower (0.74 ± 0.29 mg vs. 1.72 ± 0.63 mg, *p* < 0.001) and the stimulation duration was significantly shorter (10.78 ± 2.25 vs. 11.58 ± 2.79, *p* = 0.001) in Group 1 than in Group 2, while the total gonadotropin dose did not significantly differ between the groups. Furthermore, the LH, E_2_, and progesterone levels on the trigger day showed no significant difference between the two groups. Meanwhile, the number of retrieved oocytes, fertilized oocytes, and available embryos did not significantly differ between the two groups, and the number of cycles canceled due to pre-ovulation did not significantly differ between groups 1 and 2 (1.91% [4/209] vs. 0.44% [1/225], *p* = 0.201).

**Table 2:** Parameters of ovarian stimulation between Group 1 and Group 2.

**Groups**	**Group 1 (*n* = 209)**	**Group 2 (*n* = 225)**	***p***
Stimulation duration (day)	10.78 ± 2.25	11.58 ± 2.79	0.001
Total Gn (IU)	2449.5 ± 1081.5	2407.5 ± 944.25	0.670
Total Cetrorelix (mg)	0.74 ± 0.29	1.72 ± 0.63	<0.001
E_2_ level on hCG day (pg/ml)	5212.23 ± 2330.34	5056.78 ± 2521.74	0.517
P_4_ level on hCG day (ng/ml)	1.44 ± 0.89	1.44 ± 1.14	0.979
LH level on hCG day (IU/L)	1.75 ± 1.32	1.95 ± 1.53	0.155
Number of oocytes retrieved	11.11 ± 4.95	11.00 ± 4.36	0.805
Number of fertilized oocytes	8.34 ± 4.66	8.27 ± 4.29	0.864
Number of cleavage	8.23 ± 4.70	8.12 ± 4.28	0.807
Number of available embryos	4.69 ± 3.35	4.67 ± 3.12	0.930
Number of cycles canceled for pre-ovulation	4/209 (1.91%)	1/225 (0.44%)	0.201

*Data expressed as mean ± SD, or number (percentage)*.

As shown in Table 3, there was a total of 219 fresh embryo transfer cycles: 105 in Group 1 and 114 in Group 2. There was no statistically significant difference in the average number (1.90 ± 0.29 vs. 1.86 ± 0.34, *p* > 0.05) and score (7.34 ± 0.96 vs. 7.18 ± 0.96, *p* > 0.05) of transferred embryos. The implantation rate (29.0 vs. 23.0%), clinical pregnancy rate (45.7 vs. 35.1%), ongoing pregnancy rate (39.0 vs. 28.1%), and multiple pregnancy rate (9.5 vs. 7.9%) were slightly higher in Group 1 than in Group 2, but the differences were not significant. The incidence of OHSS was not analyzed because some of the patients did not received fresh embryos in order to avoid OHSS.

**Table 3:** Clinical outcomes between Group 1 and Group 2.

**Groups**	**Group 1**	**Group 2**	***p***
Number of cycles transferred	105	114	-
Number of embryos transferred	1.90 ± 0.29	1.86 ± 0.34	0.401
Average score of embryos transferred	7.34 ± 0.96	7.18 ± 0.96	0.109
Implantation rate	58/200 (29.0%)	49/213(23.0%)	0.178
Clinical pregnancy	48/105 (45.7%)	40/114(35.1%)	0.129
Ongoing pregnancy	41/105(39.0%)	32/114(28.1%)	0.088
Multiple pregnancy	10/105(9.5%)	9/114(7.9%)	0.811

*Data expressed as mean ± SD, or number (percentage)*.

Of the 209 patients in Group 1, 30 (14.35%) were placed in subgroup 1. Patients from subgroup 1 had significantly higher basal serum FSH (8.38 ± 1.95 IU/L vs. 7.32 ± 1.82 IU/L, *p* = 0.004) and significantly lower AFC (9.5 ± 4.54 vs. 13.51 ± 5.62, *p* < 0.001) than patients from subgroup 2. Furthermore, the average age, BMI and the basal serum LH and E_2_ levels did not significantly differ between the two subgroups. Meanwhile, patients from subgroup 1 were administered higher doses of gonadotropin (2904.75 ± 1333.26 IU vs. 2372.77± 1017.64 IU, *p* = 0.012) and Cetrorelix (0.92 ± 0.32 vs. 0.72 ± 0.28 mg, *p* < 0.001) than patients from subgroup 2, while the duration of stimulation in the two subgroups remained the same. The LH level was significantly higher on the GnRH-ant start day in subgroup 1 than in subgroup 2 (8.36 ± 0.81 vs. 4.57 ± 2.74 IU/L, *p* < 0.001), while the E_2_ level was lower (1451.20 ± 635.17 vs. 2303.92± 1766.21 pg/ml, *p* < 0.001). The progesterone and LH levels on the trigger day did not significantly differ between the two subgroups, but the E_2_ level on the trigger day was significantly lower in subgroup 1 than in subgroup 2 (2956.33 ± 1451.95 vs. 5590.32 ± 2236.25 pg/ml, *p* < 0.001). Furthermore, the number of cycles canceled for pre-ovulation were significantly higher (10% [3/30] vs. 0.56% [1/179], *p* = 0.01), while the number of retrieved oocytes (6.83 ± 3.28 vs. 11.83 ± 4.82, *p* < 0.001) and available embryos were significantly lower (2.93 ± 1.86 vs. 4.99 ± 3.46, *p* < 0.001) in subgroup 1 than in subgroup 2 (Table 4).

**Table 4:** Demographic characteristics and ovarian stimulating parameters between subgroup.1 and subgroup 2.

	**Subgroup 1(*n* = 30)**	**Subgroup 2 (*n* = 179)**	***p***
Age (years)	31.07 ± 5.42	29.73 ± 4.12	0.118
BMI	20.95 ± 3.11	21.97 ± 3.48	0.131
Basal FSH level (IU/L)	8.38 ± 1.95	7.32 ± 1.82	0.004
Basal LH level (IU/L)	5.23 ± 2.68	4.44 ± 2.19	0.081
Basal E_2_ level (pg/ml)	37.33 ± 20.72	33.19 ± 14.51	0.178
Stimulating duration (day)	11.1 ± 2.34	10.72 ± 2.24	0.394
Total Gn used (IU)	2904.75 ± 1333.26	2372.77 ± 1017.64	0.012
Total Cetrotide used (mg)	0.92 ± 0.32	0.72 ± 0.28	<0.001
LH on start day of Cetrotide (IU/L)	8.36 ± 0.81	4.57 ± 2.74	<0.001
P_4_ on start day of Cetrotide (ng/ml)	1.01 ± 0.54	0.92 ± 0.44	0.361
E_2_ on start day of Cetrotide (pg/ml)	1451.20 ± 635.17	2303.92 ± 1766.21	<0.001
LH level on hCG day (IU/L)	2.01 ± 1.19	1.71 ± 1.34	0.255
P_4_ on hCG day (ng/ml)	1.49 ± 0.78	1.43 ± 0.91	0.739
E_2_ on hCG day (pg/ml)	2956.33 ± 1451.95	5590.32 ± 2236.25	<0.001
Antral follicle count	9.5 ± 4.54	13.51 ± 5.62	<0.001
Number of oocytes retrieved	6.83 ± 3.28	11.83 ± 4.82	<0.001
Number of fertilized oocytes	5.17 ± 3.01	8.88 ± 4.68	<0.001
Number of cleavage	5.07 ± 2.97	8.76 ± 4.73	<0.001
Number of available embryos	2.93 ± 1.86	4.99 ± 3.46	<0.001
Number of cycles canceled for pre-ovulation	3/30 (10%)	1/179 (0.56%)	0.010

*Data expressed as mean ± SD, or number (percentage)*.

## Discussion

Premature LH surge is defined as an LH level ≥10 IU/L and progesterone level ≥1.0 ng/ml, and is caused by the recruitment of multiple follicles and rapid increase in the estrogen level (20, 21). During COS in IVF, premature LH surge is harmful to ovum development, causing luteinization and untimely ovulation, leading to poor outcomes, even requiring cancellation of the cycle (3). GnRH-ant competes with native GnRH for GnRH receptor-binding sites, resulting in a direct, dose-dependent, and quickly reversible block of LH release, which plays a key role in the GnRH-ant protocol during ovarian stimulation in IVF. Although it is still controversial, the pregnancy rate achieved with GnRH-ant protocol has been considered to be lower than that achieved with GnRH-agonist protocol, and the impaired endometrial receptivity has been thought of the main cause for this difference. Doctors in reproductive health services have attempted to achieve satisfactory pregnancy outcomes by reducing the GnRH-ant dose. However, the minimum suitable daily dose of GnRH-ant remains controversial. Currently, 0.25 mg of Cetrorelix per day from day 6 of stimulation is considered the standard GnRH-ant protocol, and the LH level can be maintained within the safe range with this protocol. Some other studies have reported that reducing the GnRH-ant dose to 0.125–0.2 mg per day is also effective. We consider that the daily GnRH-ant dose requirement for the GnRH-ant protocol should be individual. Therefore, we attempted to reduce the initial daily Cetrorelix dose to 0.125 mg referring to the previous studies (14) and adjust to 0.25 mg individually according to the LH level during COS as Group 1, and patients in Group 2 received 0.25 mg Cetrorelix per day throughout the treatment duration.

First, we found there were no differences in the LH, E_2_, and progesterone levels on the trigger day, neither in terms of the number of retrieved oocytes, fertilized oocytes, and available embryos between the two groups. These results demonstrated that the initial 0.125 mg dose per day with a subsequent increase to 0.25 mg when the LH level was >10 IU/L was as effective as a GnRH-ant protocol with a consistent dose of 0.25 mg per day. Compared with the previous study (14), our study showed that adjusting the GnRH-ant dose from 0.125 to 0.25 mg per day individually according to the LH level is safer.

Furthermore, Group 1 had a notable reduction in GnRH-ant consumption and reduced duration of stimulation compared with Group 2; this might result in improved pregnancy outcomes in Group 1. Many studies have reported that GnRH-ant is unfavorable for endometrial receptivity (4, 22), and our previous studies also found that antagonists caused uterine natural killer (uNK) cells and inflammatory factors such as perforin and tumor necrosis factor α (TNFα) to increase in a dose-dependent manner (6, 7). Furthermore, prolonged ovarian stimulation was associated with a decreased rate of superior-quality embryos and lower implantation, and live birth rates (23–25). In this present study, the implantation rate, clinical pregnancy rate, and ongoing pregnancy rate were all higher in Group 1 than in Group 2, although this difference was not statistically significant. We assumed that this may be because the reduction in the GnRH-ant dose to 0.125 mg per day was not sufficient to significantly increase the implantation rate. In addition, insufficient number of cases of this retrospective study could be another reason. A multicenter randomized controlled study will be performed in the next step.

Second, there was no significant difference in the BMI between subgroups 1 and 2, suggesting that the BMI might not be an important impact factor for the selection of the GnRH-ant dose. Although a few studies have suggested that it is appropriate to reduce the GnRH-ant dose for slim patients (12), Engel et al. demonstrated that body weight did not influence the plasma concentration of Cetrorelix, and they suggested that it was not necessary to modify the dose for individuals with different body weights during COS (26). Hsieh et al. suggested that reducing the Cetrorelix dose is not the proper indication even in patients weighing <50 kg (11). These studies supported our opinion that the BMI is not an important impact factor when selecting the GnRH-ant dose.

In addition, compared with subgroup 2, patients in subgroup 1 had significantly higher basal serum FSH level and lower AFCs. Furthermore, patients in subgroup 1 had a higher gonadotropin requirement, higher LH level, and lower E_2_ level at the start of the Cetrorelix treatment, indicating that subgroup 1 patients had lower ovarian reserve than subgroup 2 patients. Common indicators used to evaluate ovarian reserve include the basal FSH, AFC, anti-Müllerian hormone (AMH), and age (27). Basal FSH levels >8 IU/L (28) and a total AFC < 11 (29) in women often predicts poor ovarian response and lower pregnancy rates. In our study, the average basal FSH were higher, and AFCs were more less in subgroup 1 than in subgroup 2. The patients in subgroup 1 were slightly older than those in subgroup 2, but the difference was not statistically significant. This may be because we excluded patients over 45 years of age and patients who had an LH value >10 IU/L at the GnRH-ant start day. Meanwhile, more gonadotropin was consumed in subgroup 1 than in subgroup 2. Together, these results indicated that the ovarian response was lower in subgroup 1 than in subgroup 2. Furthermore, a higher percentage of cycles was canceled for pre-ovulation in subgroup 1 than in subgroup 2 (10 vs. 0.56%, *p* = 0.01), suggesting that patients with lower ovarian reserve were prone to early LH rise and pre-ovulation, and need sufficient GnRH antagonists from the beginning. Many studies have demonstrated that patients with lower ovarian reserve are prone to present a premature LH surge (30) and that the follicles of these patients biologically “matured” quickly and were prone to premature luteinization (31, 32). Reichman et al. declared that the diminished ovarian reserve was a predominant risk factor for GnRH-ant failure in IVF cycles (33). Another study reported that people with multiple follicles hyper-secrete putative gonadotropin surge-attenuating factor, which can attenuate the LH increase (34). We speculated that patients with a lower ovarian reserve might secrete less gonadotropin surge-attenuating factor and exhibit high pituitary sensitivity to serum hormones, and thereby may be more susceptible LH elevation and breakthrough of ovulation.

We did not compare the pregnancy outcomes between the two subgroups, because subgroup 1 only had 30 patients and embryo transfer was canceled in some of these patients. AMH is a new powerful indicator for ovarian reserve, but we did not involve this in the analysis because AMH was not performed as a routine examination due to economic reasons, and AMH data were not available.

## Conclusion

In conclusion, the results of the present study revealed that for patients with sufficient ovarian reserve, a flexible low-dose GnRH-ant protocol starting at 0.125 mg per day is as effective as a fixed GnRH-ant protocol with 0.25 mg per day. The retrospective analysis and the small sample size are the main limitations of this study, and a large sample size RCT will be conducted in the next step.

## Ethics Statement

This retrospective analysis was approved by the Institutional Review Board and the Institutional Ethics Committee of Ruijin Hospital (Research Ethics Committee reference number: 2012-57).

## Author Contributions

DZ conducted the analysis and wrote the manuscript. LX collected the data. HX statistically analyzed the data. QC and BJ was involved in patient recruitment and treatment. AZ supervised the study concept and reviewed the manuscript. BX conceived the analysis, was involved in the patient's treatment, and reviewed the manuscript.

### Conflict of Interest Statement

The authors declare that the research was conducted in the absence of any commercial or financial relationships that could be construed as a potential conflict of interest.
