# Supplementary material for: Flexible Low-Dose GnRH Antagonist Protocol Is Effective in Patients With Sufficient Ovarian Reserve in IVF
Source: Front Endocrinol (Lausanne). 2018 Dec 19;9:767. doi: 10.3389/fendo.2018.00767 (PMC6305714; doi:10.3389/fendo.2018.00767)
Supplement: Supplementary file 2 [file Data_Sheet_2.doc]

T-TEST

[DataSet 1] Group1 vs. Group2


Group Statistics	
	Group	N	Mean	Std. Deviation	Std. Error Mean	
Age	dimension1	1	209	29.92	4.342	.300	
		2
	225	30.48	4.410	.294	
BMI	dimension1	1	209	21.824545	3.4393411	.2379042	
		2	225	21.893391	3.7387859	.2492524	
bFSH	dimension1	1	209	7.472823	1.8712877	.1294397	
		2	225	7.393556	1.7974714	.1198314	
bLH	dimension1	1	209	4.556364	2.2780348	.1575750	
		2	225	4.312133	2.0116608	.1341107	
bE2	dimension1	1	209	33.784689	15.5633493	1.0765394	
		2	225	34.400000	17.0749399	1.1383293	
Total Cetrorelix	dimension1	1	209	.74462	.294252	.020354	
		2	225	1.72333	.633742	.042249	
Total Gn(ampoule)	dimension1	1	209	32.656	14.4164	.9972	
		2	225	32.102	12.5866	.8391	
stimulation duration	dimension1	1	209	10.78	2.252	.156	
		2	225	11.58	2.794	.186	
LH on trigger day	dimension1	1	209	1.7502	1.32436	.09161	
		2	225	1.9460	1.52521	.10168	
E2 on trigger day	dimension1	1	209	5212.23	2330.342	161.193	
		2	225	5060.78	2521.739	168.116	
P on trigger day	dimension1	1	209	1.4373	.89286	.06176	
		2	225	1.4399	1.14016	.07601	
AFC	dimension1	1	209	12.94	5.645	.390	
		2	225	12.73	5.648	.377	
Oocyte retrived	dimension1	1	209	11.11	4.946	.342	
		2	225	11.00	4.360	.291	
Fertilized	dimension1	1	209	8.34	4.655	.322	
		2	225	8.27	4.290	.286	
Cleavage	dimension1	1	209	8.23	4.695	.325	
		2	225	8.12	4.277	.285	
Available embryos	dimension1	1	209	4.69	3.350	.232	
		2	225	4.67	3.117	.208	
No. of transferred embryos	dimension1	1	105	1.90	.295	.029	
		2	114	1.87	.340	.032	


Independent Samples Test	
	Levene's Test for Equality of Variances	T-test for Equality of Means	
	F	Sig.	t	df	
Age	Equal variances assumed	.210	.647	-1.345	432	
	Equal variances not assumed			-1.346	430.528	
BMI	Equal variances assumed	.041	.839	-.199	432	
	Equal variances not assumed			-.200	431.961	
bFSH	Equal variances assumed	.260	.610	.450	432	
	Equal variances not assumed			.449	426.449	
bLH	Equal variances assumed	.281	.596	1.186	432	
	Equal variances not assumed			1.180	415.857	
bE2	Equal variances assumed	1.481	.224	-.391	432	
	Equal variances not assumed			-.393	431.849	
Total Cetrorelix	Equal variances assumed	13.523	.000	-20.378	432	
	Equal variances not assumed			-20.870	321.397	
Total Gn(ampoule)	Equal variances assumed	3.707	.055	.427	432	
	Equal variances not assumed			.425	414.068	
stimulation duration	Equal variances assumed	.283	.595	-3.298	432	
	Equal variances not assumed			-3.324	423.675	
LH on trigger day	Equal variances assumed	5.023	.026	-1.423	432	
	Equal variances not assumed			-1.430	430.072	
E2 on trigger day	Equal variances assumed	1.838	.176	.648	432	
	Equal variances not assumed			.650	431.989	
P on trigger day	Equal variances assumed	.408	.523	-.027	432	
	Equal variances not assumed			-.027	420.176	
AFC	Equal variances assumed	.000	.993	.377	432	
	Equal variances not assumed			.377	429.685	
Oocyte retrived	Equal variances assumed	3.126	.078	.247	432	
	Equal variances not assumed			.246	415.587	
Fertilized	Equal variances assumed	.272	.602	.171	432	
	Equal variances not assumed			.170	421.847	
Cleavage	Equal variances assumed	.367	.545	.244	432	
	Equal variances not assumed			.243	420.355	
Available embryos	Equal variances assumed	.828	.363	.087	432	
	Equal variances not assumed			.087	423.011	
No. of transferred embryos	Equal variances assumed	2.881	.091	.842	217	
	Equal variances not assumed			.847	216.276	

Independent Samples Test	
	T-test for Equality of Means	
	Sig. (2-tailed)	Mean Difference	Std. Error Difference	
Age	Equal variances assumed	.179	-.566	.421	
	Equal variances not assumed	.179	-.566	.420	
BMI	Equal variances assumed	.842	-.0688460	.3456273	
	Equal variances not assumed	.842	-.0688460	.3445652	
bFSH	Equal variances assumed	.653	.0792674	.1761302	
	Equal variances not assumed	.653	.0792674	.1763922	
bLH	Equal variances assumed	.236	.2442303	.2059754	
	Equal variances not assumed	.239	.2442303	.2069192	
bE2	Equal variances assumed	.696	-.6153110	1.5721182	
	Equal variances not assumed	.695	-.6153110	1.5667580	
Total Cetrorelix	Equal variances assumed	.000	-.978716	.048029	
	Equal variances not assumed	.000	-.978716	.046897	
Total Gn(ampoule)	Equal variances assumed	.670	.5537	1.2968	
	Equal variances not assumed	.671	.5537	1.3033	
stimulation duration	Equal variances assumed	.001	-.807	.245	
	Equal variances not assumed	.001	-.807	.243	
LH on trigger day	Equal variances assumed	.155	-.19576	.13757	
	Equal variances not assumed	.153	-.19576	.13686	
E2 on trigger day	Equal variances assumed	.517	151.452	233.587	
	Equal variances not assumed	.516	151.452	232.908	
P on trigger day	Equal variances assumed	.979	-.00264	.09881	
	Equal variances not assumed	.979	-.00264	.09794	
AFC	Equal variances assumed	.706	.204	.542	
	Equal variances not assumed	.706	.204	.542	
Oocyte retrived	Equal variances assumed	.805	.110	.447	
	Equal variances not assumed	.806	.110	.449	
Fertilized	Equal variances assumed	.864	.073	.429	
	Equal variances not assumed	.865	.073	.431	
Cleavage	Equal variances assumed	.807	.105	.431	
	Equal variances not assumed	.808	.105	.432	
Available embryos	Equal variances assumed	.930	.027	.310	
	Equal variances not assumed	.931	.027	.311	
No. of transferred embryos	Equal variances assumed	.401	.036	.043	
	Equal variances not assumed	.398	.036	.043	

Independent Samples Test	
	T-test for Equality of Means	
	95% Confidence Interval of the Difference	
	Lower	Upper	
Age	Equal variances assumed	-1.392	.261	
	Equal variances not assumed	-1.392	.260	
BMI	Equal variances assumed	-.7481661	.6104742	
	Equal variances not assumed	-.7460789	.6083869	
bFSH	Equal variances assumed	-.2669113	.4254461	
	Equal variances not assumed	-.2674389	.4259737	
bLH	Equal variances assumed	-.1606083	.6490689	
	Equal variances not assumed	-.1625076	.6509682	
bE2	Equal variances assumed	-3.7052630	2.4746410	
	Equal variances not assumed	-3.6947307	2.4641087	
Total Cetrorelix	Equal variances assumed	-1.073115	-.884317	
	Equal variances not assumed	-1.070979	-.886453	
Total Gn(ampoule)	Equal variances assumed	-1.9951	3.1025	
	Equal variances not assumed	-2.0081	3.1156	
stimulation duration	Equal variances assumed	-1.288	-.326	
	Equal variances not assumed	-1.284	-.330	
LH on trigger day	Equal variances assumed	-.46615	.07463	
	Equal variances not assumed	-.46476	.07324	
E2 on trigger day	Equal variances assumed	-307.656	610.561	
	Equal variances not assumed	-306.322	609.226	
P on trigger day	Equal variances assumed	-.19685	.19157	
	Equal variances not assumed	-.19515	.18987	
AFC	Equal variances assumed	-.862	1.271	
	Equal variances not assumed	-.862	1.271	
Oocyte retrived	Equal variances assumed	-.768	.989	
	Equal variances not assumed	-.772	.993	
Fertilized	Equal variances assumed	-.771	.917	
	Equal variances not assumed	-.773	.920	
Cleavage	Equal variances assumed	-.741	.952	
	Equal variances not assumed	-.744	.955	
Available embryos	Equal variances assumed	-.583	.637	
	Equal variances not assumed	-.585	.639	
No. of transferred embryos	Equal variances assumed	-.049	.121	
	Equal variances not assumed	-.048	.121	


[DataSet 2] Group 1 vs. Group2

Group Statistics	
	Group	N	Mean	Std. Deviation	Std. Error Mean	
Score of transferred embyros		1	200	7.34	.963	.068	
		2	213	7.18	.956	.066	


Independent Samples Test	
	Levene's Test for Equality of Variances	T-test for Equality of Means	
	F	Sig.	t	df	
Score of transferred embyros	Equal variances assumed	2.514	.114	1.608	411	
	Equal variances not assumed			1.607	408.986	

Independent Samples Test	
	T-test for Equality of Means	
	Sig. (2-tailed)	Mean Difference	Std. Error Difference	
Score of transferred embyros	Equal variances assumed	.109	.152	.094	
	Equal variances not assumed	.109	.152	.094	

Independent Samples Test	
	T-test for Equality of Means	
	95% Confidence Interval of the Difference	
	Lower	Upper	
Score of transferred embyros	Equal variances assumed	-.034	.338	
	Equal variances not assumed	-.034	.338	

[DataSet 3] Analysis of infertility type

Case Processing Summary	
	Cases	
	Valid	Missing	Total	
	N	Percent	N	Percent	N	Percent	
Primary * Group	434	100.0%	0	.0%	434	100.0%	


Primary* Group Crosstabulation	
Count	
	Group	Total	
	1	2		
Primary	0	140	148	288	
	1	69	77	146	
Total	209	225	434	


Chi-Square Tests	
	Value	df	Asymp. Sig. (2-sided)	Exact Sig. (2-sided)	Exact Sig.(1-sided)	
Pearson Chi-Square	.071a	1	.790			
Continuity Correctionb	.027	1	.869			
Likelihood Ratio	.071	1	.790			
Fisher's Exact Test				.839	.435	
Linear-by-Linear Association	.071	1	.790			
N of Valid Cases	434					
a. 0 cells (.0%) have expected count less than 5.The minimum expected count is 70.31.	
b. Computed only for a 2x2 table	


[DataSet 4] Analysis for infertility causes

1.	Male factor
Case Processing Summary	
	Cases	
	Valid	Missing	Total	
	N	Percent	N	Percent	N	Percent	
Male * Group	434	100.0%	0	.0%	434	100.0%	


Male* Group Crosstabulation	
Count	
	Group	Total	
	1	2		
Male	0	108	112	220	
	1	101	113	214	
Total	209	225	434	


Chi-Square Tests	
	Value	df	Asymp. Sig. (2-sided)	Exact Sig. (2-sided)	Exact Sig.(1-sided)	
Pearson Chi-Square	.156a	1	.693			
Continuity Correctionb	.089	1	.765			
Likelihood Ratio	.156	1	.693			
Fisher's Exact Test				.702	.383	
Linear-by-Linear Association	.156	1	.693			
N of Valid Cases	434					
a. 0 cells (.0%) have expected count less than 5.The minimum expected count is 103.06.	
b. Computed only for a 2x2 table	


Case Processing Summary	
	Cases	
	Valid	Missing	Total	
	N	Percent	N	Percent	N	Percent	
Tubal * Group	434	100.0%	0	.0%	434	100.0%	

2.	Tubal factor

Tubal* Group Crosstabulation	
Count	
	Group	Total	
	1	2		
Tubal	0	63	72	135	
	1	146	153	299	
Total	209	225	434	


Chi-Square Tests	
	Value	df	Asymp. Sig. (2-sided)	Exact Sig. (2-sided)	Exact Sig.(1-sided)	
Pearson Chi-Square	.174a	1	.676			
Continuity Correctionb	.098	1	.754			
Likelihood Ratio	.174	1	.676			
Fisher's Exact Test				.680	.377	
Linear-by-Linear Association	.174	1	.677			
N of Valid Cases	434					
a. 0 cells (.0%) have expected count less than 5.The minimum expected count is 65.01.	
b. Computed only for a 2x2 table	


3.	Ovulation disorder

Case Processing Summary	
	Cases	
	Valid	Missing	Total	
	N	Percent	N	Percent	N	Percent	
Ovulation * Group	434	100.0%	0	.0%	434	100.0%	


Ovulation* Group Crosstabulation	
Count	
	Group	Total	
	1	2		
Ovulation	0	177	195	372	
	1	32	30	62	
Total	209	225	434	


Chi-Square Tests	
	Value	df	Asymp. Sig. (2-sided)	Exact Sig. (2-sided)	Exact Sig.(1-sided)	
Pearson Chi-Square	.346a	1	.556			
Continuity Correctionb	.203	1	.652			
Likelihood Ratio	.346	1	.556			
Fisher's Exact Test				.585	.326	
Linear-by-Linear Association	.345	1	.557			
N of Valid Cases	434					
a. 0 cells (.0%) have expected count less than 5.The minimum expected count is 29.86.	
b. Computed only for a 2x2 table	

4.	Endometriosis
Case Processing Summary	
	Cases	
	Valid	Missing	Total	
	N	Percent	N	Percent	N	Percent	
Endometriosis * Group	434	100.0%	0	.0%	434	100.0%	


Endometriosis* Group Crosstabulation	
Count	
	Group	Total	
	1	2		
Endometriosis	0	193	207	400	
	1	16	18	34	
Total	209	225	434	


Chi-Square Tests	
	Value	df	Asymp. Sig. (2-sided)	Exact Sig. (2-sided)	Exact Sig.(1-sided)	
Pearson Chi-Square	.018a	1	.894			
Continuity Correctionb	.000	1	1.000			
Likelihood Ratio	.018	1	.894			
Fisher's Exact Test				1.000	.519	
Linear-by-Linear Association	.018	1	.894			
N of Valid Cases	434					
a. 0 cells (.0%) have expected count less than 5.The minimum expected count is 16.37.	
b. Computed only for a 2x2 table	

5.	Other factors

Case Processing Summary	
	Cases	
	Valid	Missing	Total	
	N	Percent	N	Percent	N	Percent	
Other * Group	434	100.0%	0	.0%	434	100.0%	


Other* Group Crosstabulation	
Count	
	Group	Total	
	1	2		
Other	0	206	222	428	
	1	3	3	6	
Total	209	225	434	


Chi-Square Tests	
	Value	df	Asymp. Sig. (2-sided)	Exact Sig. (2-sided)	Exact Sig.(1-sided)	
Pearson Chi-Square	.008a	1	.927			
Continuity Correctionb	.000	1	1.000			
Likelihood Ratio	.008	1	.928			
Fisher's Exact Test				1.000	.622	
Linear-by-Linear Association	.008	1	.928			
N of Valid Cases	434					
a. 2 cells (50.0%) have expected count less than 5.The minimum expected count is 2.89.	
b. Computed only for a 2x2 table	

[Data Set5] Analysis for cycles cancelled for pre-ovulation between Group1 and Group2

Case Processing Summary	
	Cases	
	Valid	Missing	Total	
	N	Percent	N	Percent	N	Percent	
Cancelled * Group	434	100.0%	0	.0%	434	100.0%	


Cancelled* Group Crosstabulation	
Count	
	Group	Total	
	1	2		
Cancelled	0	205	224	429	
	1	4	1	5	
Total	209	225	434	


Chi-Square Tests	
	Value	df	Asymp. Sig. (2-sided)	Exact Sig. (2-sided)	Exact Sig.(1-sided)	
Pearson Chi-Square	2.054a	1	.152			
Continuity Correctionb	.967	1	.326			
Likelihood Ratio	2.179	1	.140			
Fisher's Exact Test				.201	.164	
Linear-by-Linear Association	2.050	1	.152			
N of Valid Cases	434					
a. 2 cells (50.0%) have expected count less than 5.The minimum expected count is 2.41.	
b. Computed only for a 2x2 table	

[DataSet 5] Analysis for pregnancy rates between Group1 and Group2
1.	Implantation rate

Case Processing Summary	
	Cases	
	Valid	Missing	Total	
	N	Percent	N	Percent	N	Percent	
Implantation * Group	413	100.0%	0	.0%	413	100.0%	


Implantation* Group Crosstabulation	
Count	
	Group	Total	
	1	2		
Implantation	0	142	164	306	
	1	58	49	107	
Total	200	213	413	


Chi-Square Tests	
	Value	df	Asymp. Sig. (2-sided)	Exact Sig. (2-sided)	Exact Sig.(1-sided)	
Pearson Chi-Square	1.931a	1	.165			
Continuity Correctionb	1.632	1	.201			
Likelihood Ratio	1.932	1	.165			
Fisher's Exact Test				.178	.101	
Linear-by-Linear Association	1.927	1	.165			
N of Valid Cases	413					
a. 0 cells (.0%) have expected count less than 5.The minimum expected count is 51.82.	
b. Computed only for a 2x2 table	

2.	Clinical pregnancy rate

Case Processing Summary	
	Cases	
	Valid	Missing	Total	
	N	Percent	N	Percent	N	Percent	
Pregnancy * Group	219	100.0%	0	.0%	219	100.0%	


Pregnancy* Group Crosstabulation	
Count	
	Group	Total	
	1	2		
Pregnancy	0	57	74	131	
	1	48	40	88	
Total	105	114	219	


Chi-Square Tests	
	Value	df	Asymp. Sig. (2-sided)	Exact Sig. (2-sided)	Exact Sig.(1-sided)	
Pearson Chi-Square	2.568a	1	.109			
Continuity Correctionb	2.145	1	.143			
Likelihood Ratio	2.571	1	.109			
Fisher's Exact Test				.129	.071	
Linear-by-Linear Association	2.556	1	.110			
N of Valid Cases	219					
a. 0 cells (.0%) have expected count less than 5.The minimum expected count is 42.19.	
b. Computed only for a 2x2 table	

3.	Ongoing pregnancy rate

Case Processing Summary	
	Cases	
	Valid	Missing	Total	
	N	Percent	N	Percent	N	Percent	
Ongoing * Group	219	100.0%	0	.0%	219	100.0%	


Ongoing* Group Crosstabulation	
Count	
	Group	Total	
	1	2		
Ongoing	0	64	82	146	
	1	41	32	73	
Total	105	114	219	


Chi-Square Tests	
	Value	df	Asymp. Sig. (2-sided)	Exact Sig. (2-sided)	Exact Sig.(1-sided)	
Pearson Chi-Square	2.964a	1	.085			
Continuity Correctionb	2.491	1	.115			
Likelihood Ratio	2.967	1	.085			
Fisher's Exact Test				.088	.057	
Linear-by-Linear Association	2.950	1	.086			
N of Valid Cases	219					
a. 0 cells (.0%) have expected count less than 5.The minimum expected count is 35.00.	
b. Computed only for a 2x2 table	

4.	Multiple pregnancy rate

Case Processing Summary	
	Cases	
	Valid	Missing	Total	
	N	Percent	N	Percent	N	Percent	
Multiple * Group	219	100.0%	0	.0%	219	100.0%	


Multiple* Group Crosstabulation	
Count	
	Group	Total	
	1	2		
Multiple	0	95	105	200	
	1	10	9	19	
Total	105	114	219	


Chi-Square Tests	
	Value	df	Asymp. Sig. (2-sided)	Exact Sig. (2-sided)	Exact Sig.(1-sided)	
Pearson Chi-Square	.183a	1	.669			
Continuity Correctionb	.035	1	.851			
Likelihood Ratio	.183	1	.669			
Fisher's Exact Test				.811	.425	
Linear-by-Linear Association	.182	1	.669			
N of Valid Cases	219					
a. 0 cells (.0%) have expected count less than 5.The minimum expected count is 9.11.	
b. Computed only for a 2x2 table	

T-TEST GROUPS=subgroups('1' '2')
T-TEST
[DataSet 6] 

Group Statistics	
	subgroups	N	Mean	Std. Deviation	Std. Error Mean	
Age	dimension1	1	30	31.07	5.420	.990	
		2	179	29.73	4.121	.308	
BMI	dimension1	1	30	20.946000	3.1071226	.5672804	
		2	179	21.971788	3.4782129	.2599738	
bFSH	dimension1	1	30	8.383000	1.9521273	.3564081	
		2	179	7.320279	1.8187940	.1359430	
bLH	dimension1	1	30	5.227333	2.6776134	.4888631	
		2	179	4.443911	2.1925764	.1638809	
bE2	dimension1	1	30	37.333333	20.7203604	3.7830029	
		2	179	33.189944	14.5125947	1.0847223	
Total Cetrorelix	dimension1	1	30	.92083	.320767	.058564	
		2	179	.71508	.279828	.020915	
Total Gn(ampoule)	dimension1	1	30	38.733	17.7767	3.2456	
		2	179	31.637	13.5686	1.0142	
stimulation duration	dimension1	1	30	11.10	2.339	.427	
		2	179	10.72	2.239	.167	
LH on trigger day	dimension1	1	30	2.0057	1.18791	.21688	
		2	179	1.7074	1.34417	.10047	
E2 on trigger day	dimension1	1	30	2956.33	1451.952	265.089	
		2	179	5590.32	2236.251	167.145	
P on trigger day	dimension1	1	30	1.4877	.78214	.14280	
		2	179	1.4288	.91181	.06815	
AFC	dimension1	1	30	9.50	4.539	.829	
		2	179	13.51	5.617	.420	
Oocyte retrived	dimension1	1	30	6.83	3.281	.599	
		2	179	11.83	4.820	.360	
Fertilized	dimension1	1	30	5.17	3.007	.549	
		2	179	8.88	4.676	.349	
Cleavage	dimension1	1	30	5.07	2.970	.542	
		2	179	8.76	4.728	.353	
Available embryos	dimension1	1	30	2.93	1.856	.339	
		2	179	4.99	3.456	.258	
LH on Ant start day 	dimension1	1	30	8.36	.814	.149	
		2	179	4.57	2.744	.205	
E2 on Ant start day	dimension1	1	30	1451.20	635.173	115.966	
		2	179	2303.92	1766.209	132.013	
P on Ant start day	dimension1	1	30	1.01	.540	.099	
		2	179	.92	.440	.033	


Independent Samples Test	
	Levene's Test for Equality of Variances	T-test for Equality of Means	
	F	Sig.	t	df	Sig. (2-tailed)	
Age	Equal variances assumed	2.768	.098	1.570	207	.118	
	Equal variances not assumed			1.293	34.840	.204	
BMI	Equal variances assumed	.668	.415	-1.517	207	.131	
	Equal variances not assumed			-1.644	42.157	.108	
bFSH	Equal variances assumed	.151	.698	2.931	207	.004	
	Equal variances not assumed			2.786	37.921	.008	
bLH	Equal variances assumed	.100	.752	1.752	207	.081	
	Equal variances not assumed			1.519	35.810	.137	
bE2	Equal variances assumed	3.478	.064	1.352	207	.178	
	Equal variances not assumed			1.053	33.927	.300	
Total Cetrorelix	Equal variances assumed	.384	.536	3.648	207	.000	
	Equal variances not assumed			3.309	36.772	.002	
Total Gn(ampoule)	Equal variances assumed	1.709	.193	2.527	207	.012	
	Equal variances not assumed			2.087	34.885	.044	
stimulation duration	Equal variances assumed	.106	.745	.853	207	.394	
	Equal variances not assumed			.827	38.437	.413	
LH on trigger day	Equal variances assumed	.160	.689	1.142	207	.255	
	Equal variances not assumed			1.248	42.463	.219	
E2 on trigger day	Equal variances assumed	9.118	.003	-6.228	207	.000	
	Equal variances not assumed			-8.405	55.220	.000	
P on trigger day	Equal variances assumed	.904	.343	.333	207	.739	
	Equal variances not assumed			.372	43.349	.712	
AFC	Equal variances assumed	2.200	.140	-3.713	207	.000	
	Equal variances not assumed			-4.321	45.312	.000	
Oocyte retrived	Equal variances assumed	5.740	.017	-5.467	207	.000	
	Equal variances not assumed			-7.152	52.649	.000	
Fertilized	Equal variances assumed	5.176	.024	-4.199	207	.000	
	Equal variances not assumed			-5.702	55.777	.000	
Cleavage	Equal variances assumed	5.587	.019	-4.139	207	.000	
	Equal variances not assumed			-5.706	57.175	.000	
Available embryos	Equal variances assumed	8.610	.004	-3.177	207	.002	
	Equal variances not assumed			-4.825	68.729	.000	
LH on Ant start day 	Equal variances assumed	48.982	.000	7.503	207	.000	
	Equal variances not assumed			14.974	153.707	.000	
E2 on Ant start day	Equal variances assumed	15.640	.000	-2.612	207	.010	
	Equal variances not assumed			-4.853	120.024	.000	
P on Ant start day	Equal variances assumed	.340	.561	.916	207	.361	
	Equal variances not assumed			.792	35.745	.434	

Independent Samples Test	
	T-test for Equality of Means	
	Mean Difference	Std. Error Difference	95% Confidence Interval of the Difference	
			Lower	Upper	
Age	Equal variances assumed	1.340	.854	-.342	3.023	
	Equal variances not assumed	1.340	1.036	-.764	3.445	
BMI	Equal variances assumed	-1.0257877	.6764076	-2.3593188	.3077433	
	Equal variances not assumed	-1.0257877	.6240140	-2.2849596	.2333841	
bFSH	Equal variances assumed	1.0627207	.3626142	.3478303	1.7776111	
	Equal variances not assumed	1.0627207	.3814541	.2904546	1.8349868	
bLH	Equal variances assumed	.7834227	.4471951	-.0982181	1.6650635	
	Equal variances not assumed	.7834227	.5156007	-.2624561	1.8293015	
bE2	Equal variances assumed	4.1433892	3.0642628	-1.8977754	10.1845538	
	Equal variances not assumed	4.1433892	3.9354458	-3.8550304	12.1418088	
Total Cetrorelix	Equal variances assumed	.205750	.056406	.094546	.316953	
	Equal variances not assumed	.205750	.062187	.079721	.331778	
Total Gn(ampoule)	Equal variances assumed	7.0965	2.8080	1.5606	12.6323	
	Equal variances not assumed	7.0965	3.4003	.1926	14.0003	
stimulation duration	Equal variances assumed	.379	.444	-.497	1.256	
	Equal variances not assumed	.379	.459	-.549	1.308	
LH on trigger day	Equal variances assumed	.29824	.26108	-.21648	.81295	
	Equal variances not assumed	.29824	.23902	-.18397	.78045	
E2 on trigger day	Equal variances assumed	-2633.985	422.917	-3467.763	-1800.208	
	Equal variances not assumed	-2633.985	313.384	-3261.965	-2006.005	
P on trigger day	Equal variances assumed	.05884	.17652	-.28917	.40685	
	Equal variances not assumed	.05884	.15823	-.26018	.37786	
AFC	Equal variances assumed	-4.014	1.081	-6.145	-1.883	
	Equal variances not assumed	-4.014	.929	-5.885	-2.143	
Oocyte retrived	Equal variances assumed	-4.999	.914	-6.802	-3.196	
	Equal variances not assumed	-4.999	.699	-6.401	-3.597	
Fertilized	Equal variances assumed	-3.710	.884	-5.453	-1.968	
	Equal variances not assumed	-3.710	.651	-5.014	-2.407	
Cleavage	Equal variances assumed	-3.693	.892	-5.452	-1.934	
	Equal variances not assumed	-3.693	.647	-4.989	-2.397	
Available embryos	Equal variances assumed	-2.055	.647	-3.331	-.780	
	Equal variances not assumed	-2.055	.426	-2.905	-1.205	
No. of transferred embryos	Equal variances assumed	.056	.073	-.090	.202	
	Equal variances not assumed	.056	.060	-.066	.178	
LH on Ant start day 	Equal variances assumed	3.793	.506	2.796	4.789	
	Equal variances not assumed	3.793	.253	3.292	4.293	
E2 on Ant start day	Equal variances assumed	-852.722	326.498	-1496.410	-209.034	
	Equal variances not assumed	-852.722	175.714	-1200.623	-504.820	
P on Ant start day	Equal variances assumed	.082	.090	-.095	.260	
	Equal variances not assumed	.082	.104	-.129	.293	


[DataSet 7] Analysis for cycles cancelled for pre-ovulation between subgroup1 and subgroup2

Case Processing Summary	
	Cases	
	Valid	Missing	Total	
	N	Percent	N	Percent	N	Percent	
Cancelled * subgroups	209	100.0%	0	.0%	209	100.0%	


Cancelled* subgroups Crosstabulation	
Count	
	subgroups	Total	
	1	2		
cancelled	0	27	178	205	
	1	3	1	4	
Total	30	179	209	


Chi-Square Tests	
	Value	df	Asymp. Sig. (2-sided)	Exact Sig. (2-sided)	Exact Sig.(1-sided)	
Pearson Chi-Square	12.200a	1	.000			
Continuity Correctionb	7.689	1	.006			
Likelihood Ratio	7.697	1	.006			
Fisher's Exact Test				.010	.010	
Linear-by-Linear Association	12.142	1	.000			
N of Valid Cases	209					
a. 2 cells (50.0%) have expected count less than 5.The minimum expected count is .57.	
b. Computed only for a 2x2 table	
